# 

**Published:** 1890-12-13

**Authors:** 


made beef-tea, &c., whereas had JOHNSTON'S BOVRIL been used in [illegible]stead, the patients would in nine case[illegible] [illegible]t of ten have gained strength to battle against the disease under which they were suffering. I have no hesitation to advise you

[illegible]e to your patients, to your convalescents, to those of your clients who have mental exertions, to use JOHNSTON'S

[illegible]hich, in a concentrated form, contains a substantial tonic and a palatable food."—J. M. BEAUSOLIEL, M.D.

[illegible]CHEMISTS, GROCERS, &c.[illegible]

THROUGHOUT

THE UNITED KINGDOM.

